# Epidemiological transition of metabolic dysfunction-associated steatotic liver disease-related chronic liver disease and cirrhosis: a comparative study between China and the global population (1990–2021)

**DOI:** 10.3389/fnut.2025.1624440

**Published:** 2025-07-15

**Authors:** Yi Tang, Changhao Gu

**Affiliations:** ^1^Cangnan Hospital of Traditional Chinese Medicine, Wenzhou, China; ^2^Cangnan Branch of Zhejiang Provincial Hospital of Chinese Medicine, Wenzhou, China

**Keywords:** metabolic dysfunction-associated steatotic liver disease, trend, incidence, mortality, prevalence, disability-adjusted life years

## Abstract

**Objectives:**

This study systematically evaluates the epidemiological transition of metabolic dysfunction-associated steatotic liver disease (MASLD)-related chronic liver diseases (CLD) and cirrhosis burden in China versus global populations (1990–2021), with emphasis on age-sex stratified disparities in incidence, prevalence, mortality, and disability-adjusted life years (DALYs). We aim to identify critical intervention windows for precision public health strategies.

**Methods:**

Using open data from the Global Burden of Disease (GBD) database from 1990 to 2021, we conducted a comparative age-period-cohort analysis between Chinese and global populations. Joinpoint regression quantified temporal trends through average annual percentage change (AAPC) with 95% confidence intervals. Multidimensional stratification by age-sex cohorts elucidated differential burden patterns across disease progression stages.

**Results:**

From 1990 to 2021, China’s age-standardized incidence rate (ASIR) increased from 495.16 per 100,000 population in 1990 to 621.18 per 100,000 in 2021 (AAPC = 0.74%). The age-standardized prevalence rate (ASPR) rose from 12,787.39 per 100,000 to 15,606.31 per 100,000 (AAPC = 0.63%). Globally, the ASIR increased from 475.18 per 100,000 to 592.78 per 100,000 (AAPC = 0.71%), while the ASPR increased from 12,084.69 per 100,000 to 15,017.46 per 100,000 (AAPC = 0.71%). China’s age-standardized mortality rate (ASMR) declined from 0.54 per 100,000 to 0.31 per 100,000 (a decrease of 42.59%, AAPC = −1.75%), and the age-standardized DALY rate (ASDR) dropped from 13.99 per 100,000 to 7.61 per 100,000 (AAPC = −1.98%). China achieved significant reductions in mortality and age-standardized DALYs, in contrast to relatively stagnant global trends. The effects of age and gender on the disease burden varied: incidence rates remained high among younger populations, while the elderly accounted for the majority of deaths. Overall disease burden was higher in males than females; however, among individuals over 65 years old, the burden was more pronounced in females compared to males.

**Conclusion:**

From 1990 to 2021, China has made progress in controlling mortality. However, the continuously rising age-standardized incidence rate (ASIR) and age-standardized prevalence rate (ASPR) highlight an urgent need for targeted metabolic risk interventions among the youth. The observed epidemiological transition—characterized by a peak in incidence among younger populations and a concentration of deaths in older adults—calls for integrated strategies including early-life lifestyle interventions and comprehensive management frameworks for comorbidities in the elderly. Given China’s large and aging population, addressing these shifting disease patterns remains a major public health challenge.

## Introduction

Metabolic dysfunction-associated steatotic liver disease (MASLD), previously termed nonalcoholic fatty liver disease (NAFLD), encompasses a spectrum of hepatic disorders ranging from simple steatosis to progressive metabolic dysfunction-associated steatohepatitis (MASH), cirrhosis, and hepatocellular carcinoma (HCC) in individuals without significant alcohol consumption (1). Nonalcoholic fatty liver disease (NAFLD) was initially defined by the presence of hepatic steatosis affecting >5% of hepatocytes, in the absence of significant alcohol consumption (typically defined as ≤20 g/day for women and ≤30 g/day for men) or other known causes of liver disease (2).

A significant shift occurred in 2020 with the proposal of the term metabolic dysfunction-associated fatty liver disease (MAFLD) and its diagnostic criteria. The diagnosis of MAFLD requires evidence of hepatic steatosis (via histology, imaging, or blood biomarkers) in conjunction with at least one of the following three criteria: overweight/obesity, type 2 diabetes mellitus (T2DM), or evidence of metabolic dysregulation. Notably, the vast majority of patients meeting the NAFLD criteria also fulfill the MAFLD diagnostic criteria (3).

Building upon this framework, a further nomenclature revision in June 2023 introduced metabolic dysfunction-associated steatotic liver disease (MASLD), reflecting evolving insights into the pathophysiology emphasizing metabolic dysfunction as the cardinal driver. The MASLD diagnostic criteria encompass a patient population substantially similar to that of MAFLD, and MASLD has officially superseded both MAFLD and NAFLD as the new, unified standard terminology (4). The updated definition stipulates that MASLD requires hepatic steatosis >5% plus ≥1 metabolic risk factor (e.g., BMI ≥ 23 kg/m^2^, T2DM), excluding other liver diseases and significant alcohol use (≤20 g/day for women, ≤30 g/day for men).

Currently representing the predominant chronic liver condition globally, NAFLD’s escalating prevalence (20–30% adult population affected) parallels the twin pandemics of obesity and type 2 diabetes mellitus (T2DM), positioning it as a critical but underprioritized global health challenge (5). The clinical trajectory of NAFLD reveals substantial heterogeneity: 20–30% of patients progress to MASH, with 2–5% developing cirrhosis and subsequent HCC (6). It is also closely associated with extrahepatic manifestations, such as chronic kidney disease, cardiovascular disease and type 2 diabetes mellitus (7, 8).

The incidence of NAFLD and NAFLD-related cirrhosis and MASH-related liver cancer is expected to increase further globally in the next decade as obesity and T2DM become more prevalent worldwide (9–11).

Despite NAFLD’s intricate associations with prevalent noncommunicable diseases (NCDs) and substantial overlap in public health management pathways, its exclusion from the World Health Organization’s Global Action Plan for NCDs has perpetuated systemic neglect (12). This institutional oversight has exacerbated low disease awareness and fragmented care delivery models. Historically, NAFLD was conceptualized as a disorder of affluence. This classification arose from its pathophysiological ties to metabolic syndrome and obesity. However, NAFLD now presents a distinct epidemiological paradox: Developing nations report a 68% surge in prevalence over two decades. This increase is driven by urbanization-induced nutritional transitions and the adoption of sedentary lifestyles (13, 14). Such a shift challenges traditional disease paradigms and necessitates a recalibration of global health priorities.

The Global Burden of Disease Study (GBD), coordinated by the Institute for Health Metrics and Evaluation at the University of Washington, systematically documents global mortality, morbidity, and health risk factors. Through comprehensive analysis of disease and injury impacts, it identifies key drivers of mortality and disability, guiding evidence-based health policy development. The database provides essential metrics including mortality rates, disease incidence, and risk factor assessments, establishing itself as a critical tool for global public health prioritization.

Although several GBD-based studies have estimated the global prevalence of metabolic dysfunction-associated steatotic liver disease (MASLD), significant knowledge gaps remain regarding its demographic and geographic variations. The multifactorial nature and heterogeneous progression of MASLD necessitate population-specific burden assessments across regions, age groups, and genders to inform targeted prevention strategies. Existing research predominantly focuses on global aggregates, with limited investigation of national disparities. China’s status as the most populous nation warrants urgent assessment of MASLD burden, yet comprehensive national-level analyses remain unavailable.

This study analyzes GBD 2021 data to compare MASLD burden between China and global averages from 1990 to 2021. Utilizing Joinpoint regression, we examine age- and sex-specific trends over this 30 years period. The findings aim to assist Chinese policymakers in evaluating MASLD burden, developing prevention strategies, and optimizing public health resource allocation.

## Methods

### Data source

This study was based on data from the Global Burden of Disease Study 2021 (GBD 2021) coordinated by the Institute for Health Metrics and Evaluation (IHME) (15). The GBD 2021 dataset contains information on 371 diseases/injuries and 88 risk factors across 204 countries and territories from 1990 to 2021 (16, 17), accessible through the Global Health Data Exchange.^1^ Data collection and validation were conducted by IHME at the University of Washington.

Given that the nomenclature transition to Metabolic Dysfunction-Associated Steatotic Liver Disease (MASLD) postdated the finalization of the Global Burden of Disease Study (GBD) 2021, the analysis utilized GBD 2021 estimates pertaining to Non-Alcoholic Fatty Liver Disease (NAFLD).

Within the GBD 2021 methodology framework, the case definitions pertain to the patient population characterized by the progressive pathological spectrum spanning from uncomplicated hepatic steatosis to advanced cirrhosis induced by persistent steatosis and its associated inflammatory processes. This spectrum encompasses:

1. NAFLD/NASH without cirrhosis: Defined by hepatic steatosis involving ≥5% of hepatocytes, verified through imaging (ultrasound, CT, or MRI) or histopathological assessment; diagnoses relying solely on biomarkers were excluded (18).
2. Compensated cirrhosis due to NASH.
3. Decompensated cirrhosis due to NASH.

Key diagnostic safeguards include:

Documented daily ethanol intake below 20 g. Comprehensive exclusion of alternative etiologies through appropriate differential diagnosis.

For most diseases and injuries, standardized analytical tools are employed to model processed data, enabling the estimation of disease burden across study populations stratified by age, sex, geographic location, and temporal trends. The data utilized in this study were generated using two principal tools: DisMod-MR, a Bayesian meta-regression framework, and the Cause of Death Ensemble model (CODEm). DisMod-MR integrates heterogeneous epidemiological data—including incidence, prevalence, remission, and mortality rates—while ensuring internal consistency among these parameters (19). CODEm, a systematic modeling platform, employs an ensemble of predictive algorithms to analyze cause-specific mortality data, incorporating covariates that demonstrate optimal out-of-sample predictive validity (20, 21).

Leveraging the Global Burden of Disease (GBD) database, we extracted age-standardized metrics—incidence, prevalence, mortality, and disability-adjusted life years (DALYs)—for nonalcoholic fatty liver disease (NAFLD)-related cirrhosis and chronic liver diseases in China and globally (1990–2021). These indicators served as the foundation for assessing the disease burden attributable to MASLD. Given the use of publicly available GBD 2021 data, this study was exempt from institutional ethics review. All analyses adhered to the Guidelines for Accurate and Transparent Health Estimates Reporting (GATHER) to ensure methodological rigor and reproducibility.

### Statistical analysis

From the Global Burden of Disease (GBD) database, we extracted data on incidence, prevalence, mortality, and disability-adjusted life years (DALYs), along with their corresponding age-standardized rates (ASRs): Age-standardized incidence rate (ASIR); Age-standardized prevalence rate (ASPR); Age-standardized mortality rate (ASMR); Age-standardized DALYs rate (ASDR).

Additionally, we obtained crude rates (CIR, CPR, CMR, CDR) stratified by age group for both China and global populations (1990–2021).

To assess temporal trends, we computed the average annual percentage change (AAPC) and its 95% confidence interval (95% CI) using Joinpoint Regression Software (Version 5.1.0.0; National Cancer Institute, USA) (22, 23). The regression model was structured as follows: ln(y) = *α* + *β*x + *ε*: y = age-standardized metric (e.g., ASIR, ASMR); x = calendar year; β = slope coefficient.

The AAPC was derived as: AAPC = 100 × (exp(β) − 1) Trend interpretations were based on the 95% CI of AAPC: Increasing trend: 95% CI > 0; Decreasing trend: 95% CI < 0; Stable trend: 95% CI includes 0.

We specified models accommodating between 0 and 6 joinpoints to balance flexibility and parsimony. Model selection was performed using a permutation test with a significance level of *α* = 0.05. The analysis yields Annual Percent Change (APC) estimates, each accompanied by 95% confidence intervals (CI), for every identified trend segment. The Average Annual Percent Change (AAPC), representing the overall trend summary, was calculated as the weighted mean of these segment-specific APCs over the entire study period.

All statistical analyses and visualizations were conducted using R (v4.4.1) and Joinpoint (v5.1.0.0). A two-sided *p*-value < 0.05 was considered statistically significant (Table 1).

**Table 1:** Temporal trends in MASLD-related cirrhosis burden: all-age cases and age-standardized rates of incidence, prevalence, mortality, and disability-adjusted life years (DALYs) with corresponding average annual percentage change (AAPC) in China and globally, 1990–2021.

Location	Measure	1990		2021		AAPC
		All-ages cases	Age-standardized rates per 100,000 people	All-ages cases	Age-standardized rates per 100,000 people	
China	Incidence	6,179,750 (5,558,026–6,815,755)	495.16 (448.67–540.7)	9,561,143 (8,776,597–10,366,756)	621.18 (565.75–677.23)	0.74 (0.69–0.78)
	Prevalence	137,113,465 (125,346,813–151,339,953)	12,787.39 (11,678.39–14,025.33)	291,247,168 (265,154,838–317,831,032)	15,606.31 (14,271.71–17,011.02)	0.63 (0.57–0.70)
	Deaths	4,223 (2,786–5,981)	0.54 (0.35–0.76)	6,339 (4,018–9,241)	0.31 (0.2–0.45)	−1.75 (−2.03 to 1.46)
	DALYs	128,243 (83,671–181,154)	13.99 (9.07–19.73)	158,263 (102,325–231,673)	7.61 (5.01–10.94)	−1.98 (−2.27 to 1.71)
Global	Incidence	24,841,745 (22,565,086–27,319,937)	475.18 (432.27–517.76)	48,310,981 (44,191,374–52,313,165)	592.78 (542.23–643.24)	0.71 (0.69–0.73)
	Prevalence	564,415,674 (516,508,606–618,084,433)	12,084.69 (11,058.04–13,183.89)	1,267,815,566 (1,157,880,130–1,380,382,443)	15,017.46 (13,755.84–16,360.8)	0.71 (0.68–0.74)
	Deaths	44,861 (31,680–61,014)	1.16 (0.81–1.57)	97,403 (69,530–130,168)	1.14 (0.82–1.52)	−0.03 (−0.21 to 0.14)
	DALYs	1,285,242 (895,297–1,775,500)	30.57 (21.2–42.04)	2,671,793 (1,895,293–3,602,044)	30.9 (22.17–41.5)	0.04 (−0.11 to 0.19)

## Results

### Global burden of chronic liver diseases and cirrhosis attributable to MASLD

**Figure 1:**
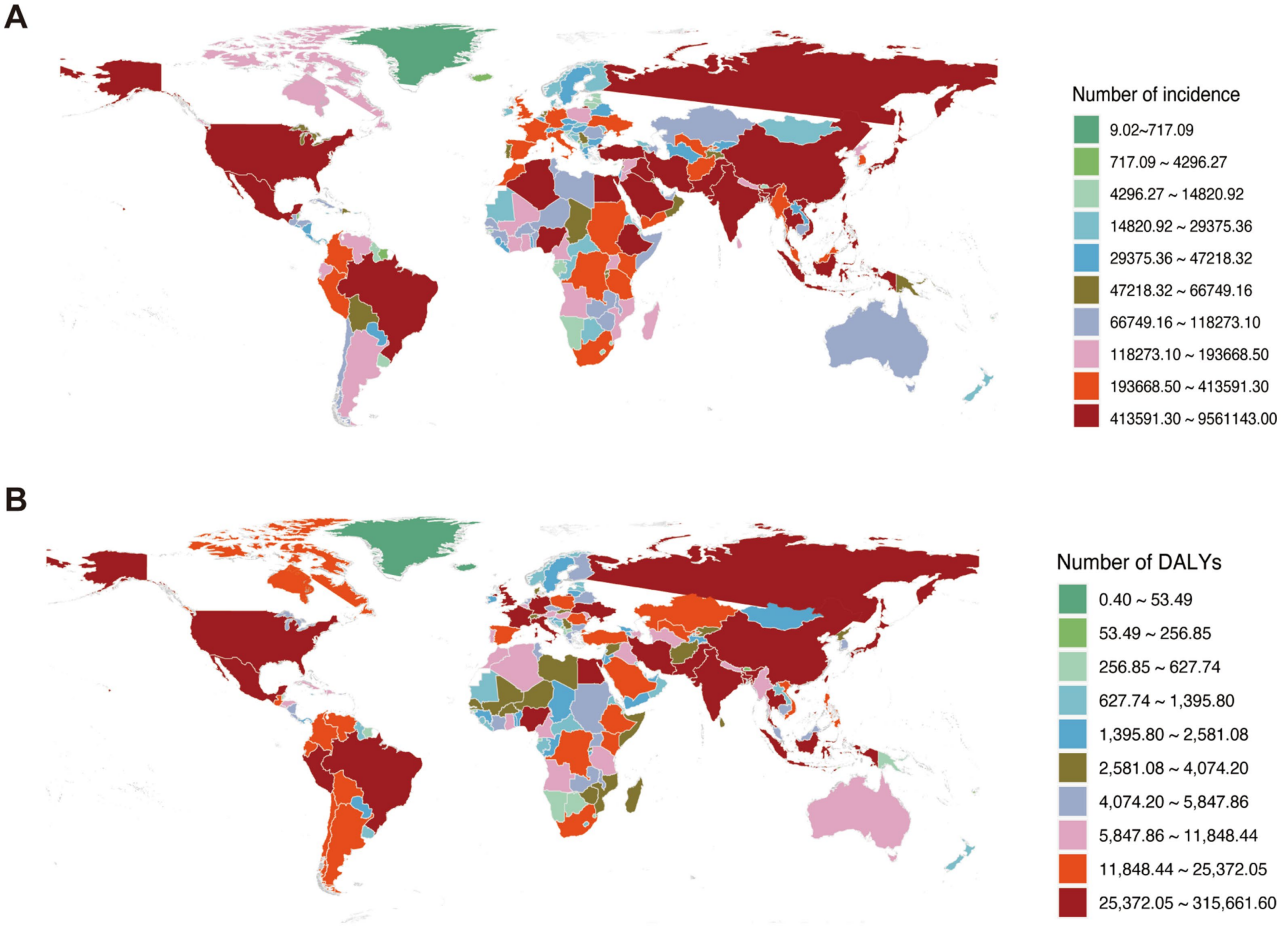
Global burden of chronic liver diseases attributable to MASLD in 2021. **(A)** Incident cases of MASLD-associated chronic liver diseases. **(B)** Disease burden measured in disability-adjusted life years (DALYs).

According to the 2021 Global Burden of Disease (GBD) data, metabolic dysfunction-associated steatotic liver disease (MASLD) imposes a substantial disease burden globally. China recorded the world’s highest incidence of MASLD-related chronic liver diseases and cirrhosis in 2021, while ranking fourth globally for related disability-adjusted life years (DALYs) (Figure 1).

### China vs. global disease burden trends (1990–2021)

#### Incidence

Cases increased 54.72% in China [6,179,750 (5,558,026–6,815,755) to 9,561,143 (8,776,597–10,366,756)] and 94.47% globally [24,841,745 (22,565,086–27,319,937) to 48,310,981 (44,191,374–52,313,165)]. Age-standardized rates rose in China [495.16 (448.67–540.70) to 621.18 (565.75–677.23) per 100,000] and globally [475.18 (432.27–517.76) to 592.78 (542.23–643.24) per 100,000]. AAPC was comparable: China 0.74 (0.69–0.78); Global 0.71 (0.69–0.73) (Table 1).

#### Prevalence

Cases increased 112.41% in China [137,113,465 (125,346,813–151,339,953) to 291,247,168 (265,154,838–317,831,032)] and 124.62% globally [564,415,674 (516,508,606–618,084,433) to 1,267,815,566 (1,157,880,130–1,380,382,443)]. Age-standardized rates rose in China [12,787.39 (11,678.39–14,025.33) to 15,606.31 (14,271.71–17,011.02) per 100,000] and globally [12,084.69 (11,058.04–13,183.89) to 15,017.46 (13,755.84–16,360.80) per 100,000]. Global AAPC was higher [0.71 (0.68–0.74)] than China’s [0.63 (0.57–0.70)] (Table 1).

#### Mortality

Global deaths increased 117.12% [44,861 (31,680–61,014) to 97,403 (69,530–130,168)] versus 50.10% in China [4,223 (2,786–5,981) to 6,339 (4,018–9,241)]. Age-standardized rates decreased globally [1.16 (0.81–1.57) to 1.14 (0.82–1.52) per 100,000] but significantly in China [0.54 (0.35–0.76) to 0.31 (0.20–0.45) per 100,000]. AAPC: Global −0.03 (−0.21–0.14); China −1.75 (−2.03 to –1.46) (Table 1).

#### DALYs

Global DALYs increased 107.88% [1,285,242 (895,297–1,775,500) to 2,671,793 (1,895,293–3,602,044)] versus 23.41% in China [128,243 (83,671–181,154) to 158,263 (102,325–231,673)]. Age-standardized rates were stable globally [30.57 (21.20–42.04) to 30.90 (22.17–41.50) per 100,000] but decreased significantly in China [13.99 (9.07–19.73) to 7.61 (5.01–10.94) per 100,000]. AAPC: Global 0.04 (−0.11 to 0.19); China −1.98 (−2.27 to −1.71) (Table 1).

### Joinpoint regression analysis (China vs. global)

**Figure 2:**
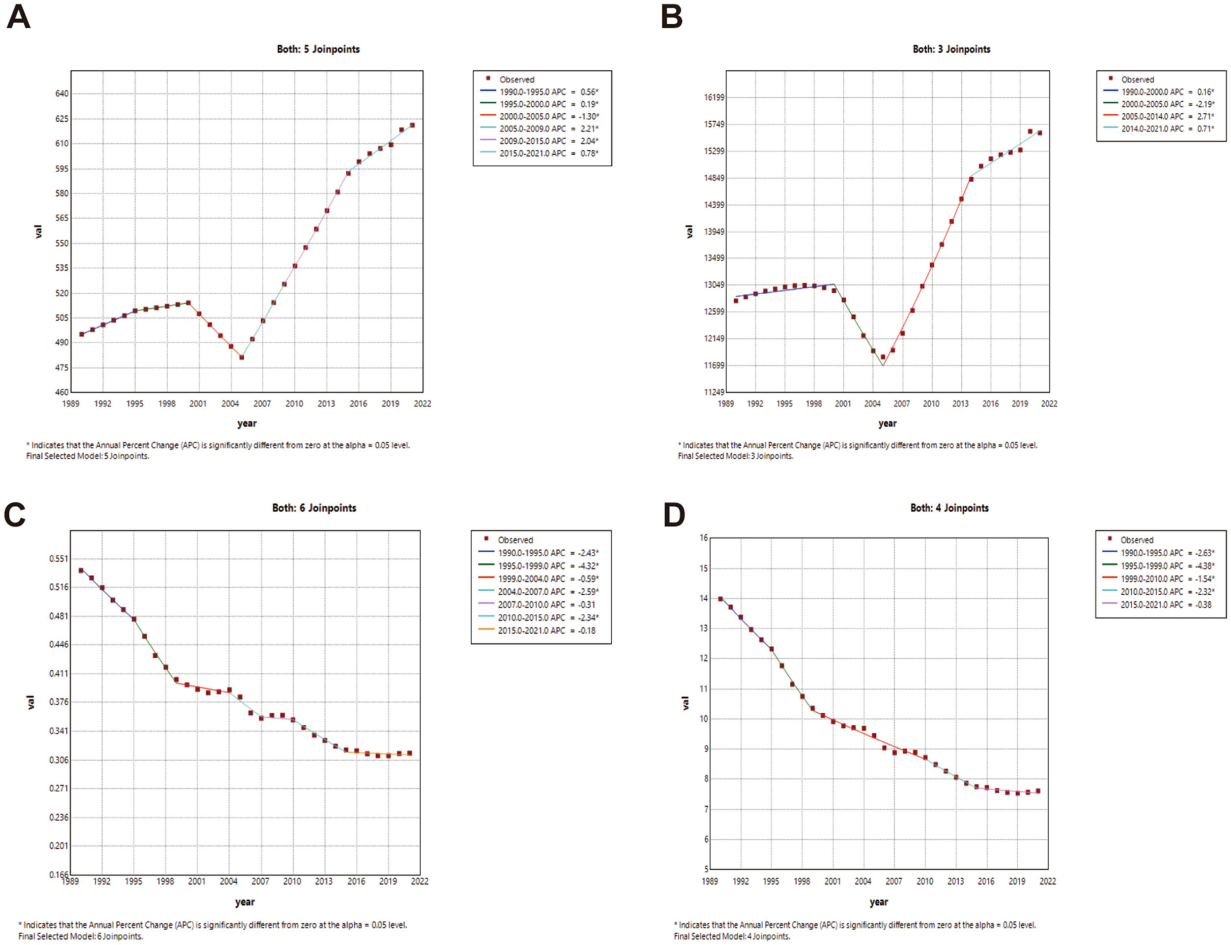
The APC of ASIR, ASPR, ASMR, and ASDR in China from 1990 to 2021 (* means *p*-values < 0.05 and significant results). **(A)** ASIR; **(B)** ASPR; **(C)** ASMR; **(D)** ASDR.

**Figure 3:**
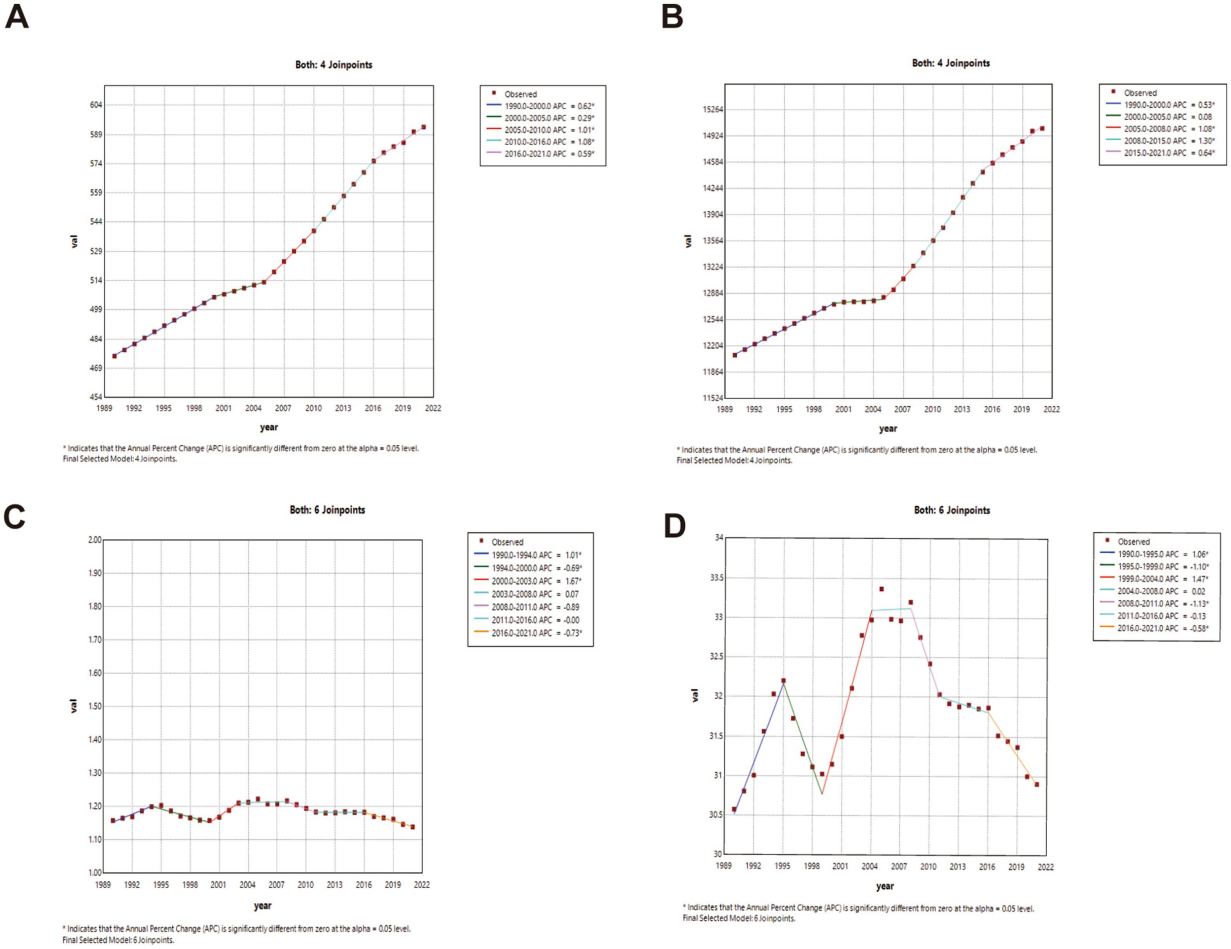
The APC of ASIR, ASPR, ASMR, and ASDR in Global from 1990 to 2021 (* means *p*-values < 0.05 and significant results). **(A)** ASIR; **(B)** ASPR; **(C)** ASMR; **(D)** ASDR.

China: ASIR and ASPR showed phased growth: gradual increase (1990–2000; ASIR APC1990–1995 = 0.56, APC1995–2000 = 0.19; ASPR APC = 0.16; *p* < 0.05), transient decline (2000–2005), then accelerated growth post-2005. ASMR declined significantly throughout (*p* < 0.05). Global: ASIR and ASPR increased significantly post-1990 (*p* < 0.05). ASMR fluctuated with minimal net change. Complete joinpoint regression analyses are presented in Figures 2, 3.

### Findings on disease burden trends

**Figure 4:**
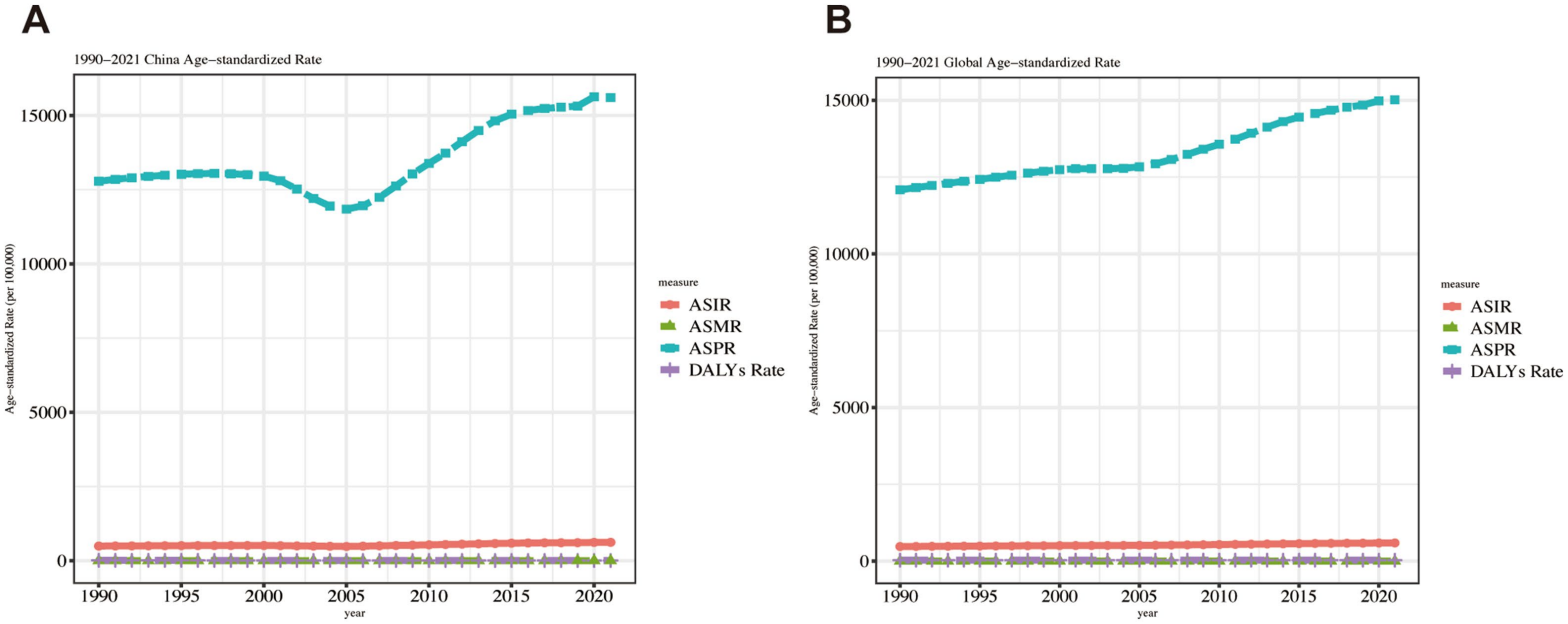
Trend comparison of ASIR, ASPR, ASMR, and ASDR in China and worldwide from 1990 to 2021.

In China, ASPR exhibited the same phased trend identified through Joinpoint regression analysis. Globally, ASPR remained stable during 2000–2005 but increased consistently in other periods. Both China and the global population demonstrated modest yet sustained ASIR increases throughout the 1990–2021 observation period. Figure 4 compares ASIR, ASPR, ASMR, and ASDR trends between China and worldwide.

### Age-specific disease burden in China (1990 vs. 2021)

**Figure 5:**
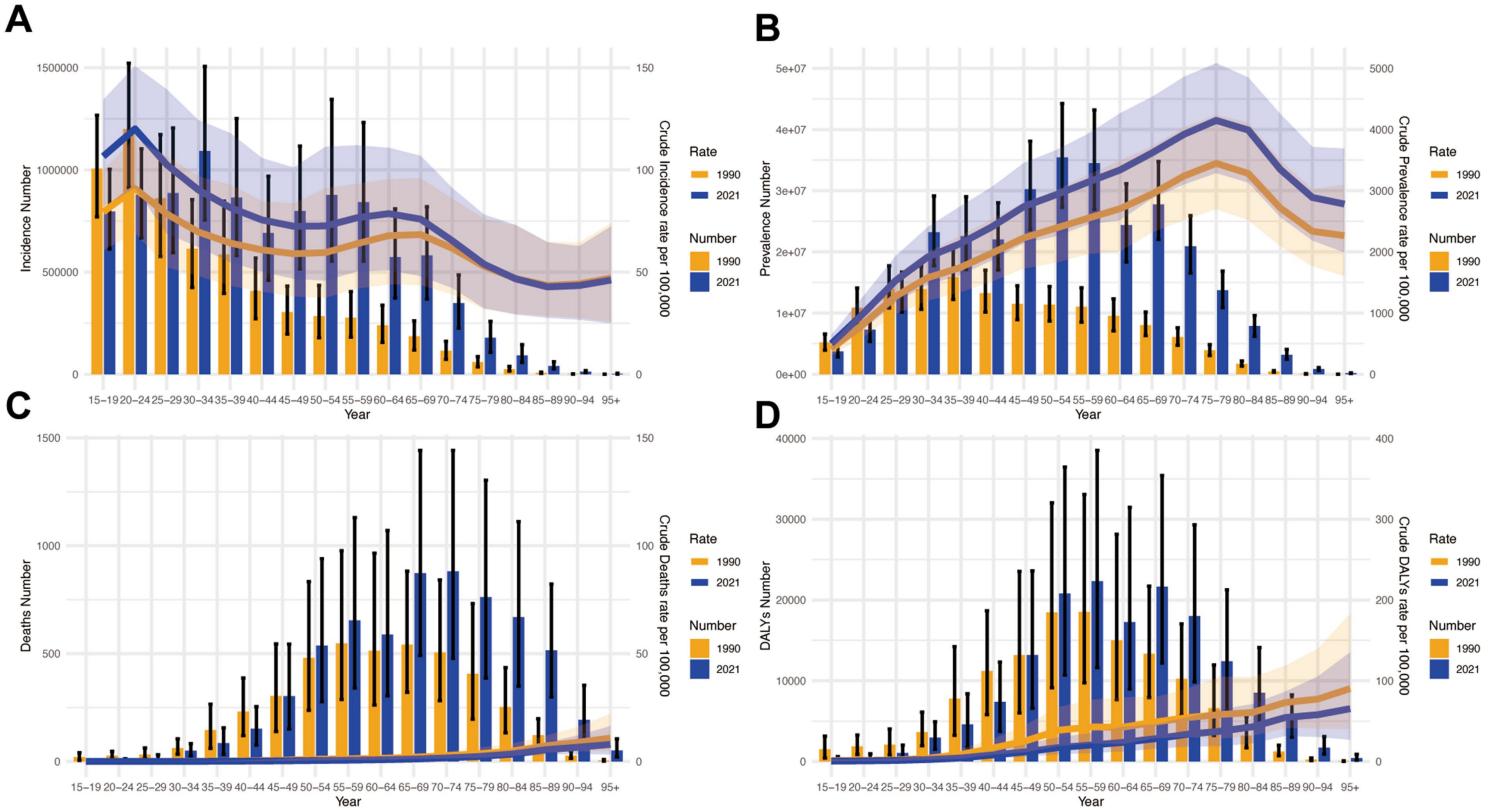
Presents a comparative analysis of disease burden indicators across age groups in China during 1990–2021. **(A)** Incidence counts with corresponding crude incidence rates (CIR); **(B)** Prevalence data showing case numbers and crude prevalence rates (CPR); **(C)** Mortality statistics including death counts and crude mortality rates (CMR); **(D)** Disability-adjusted life years (DALYs) measurements with associated crude DALYs rates (CDR).

Figure 5 presents comparative analyses of incidence, prevalence, mortality, and DALY rates across age groups in China. Key findings reveal distinct epidemiological patterns:

#### Incidence and prevalence

Highest incidence burden under 60 years, peak in 20–24 age group (Figure 5A). Prevalence peaked in 75–79 age group, showing an aging pattern (Figure 5B).

#### Mortality and DALYs

Mortality escalated with age, highest in 95+ group (Figure 5C). DALYs mirrored mortality, peaking in 95+ group (Figure 5D).

### Gender disparities in disease burden across age groups in China (1990–2021)

**Figure 6:**
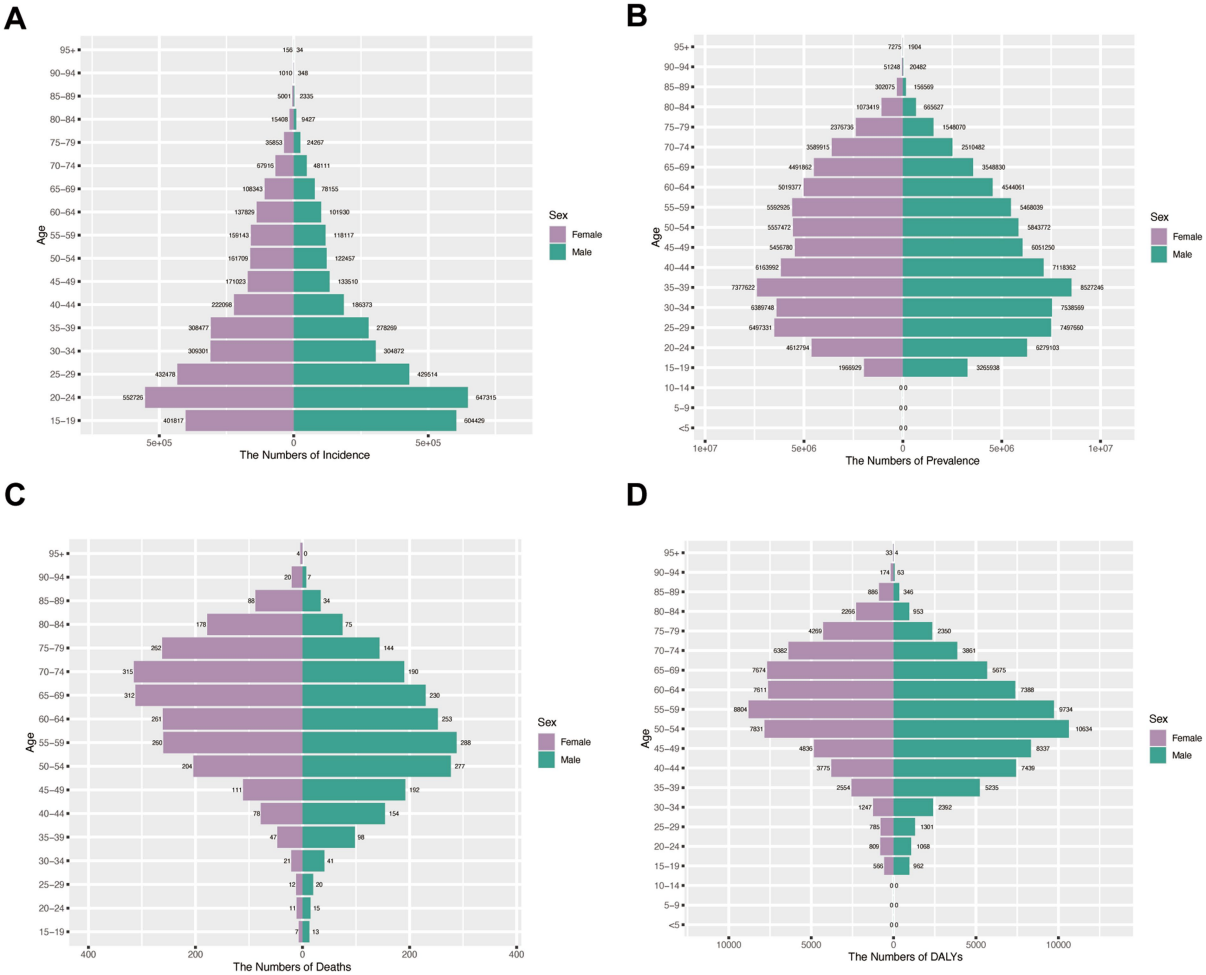
Assesses sex- and age-specific variations in burden across four metrics in China (1990). **(A)** Incidence; **(B)** Prevalence; **(C)** Mortality; **(D)** DALYs.

**Figure 7:**
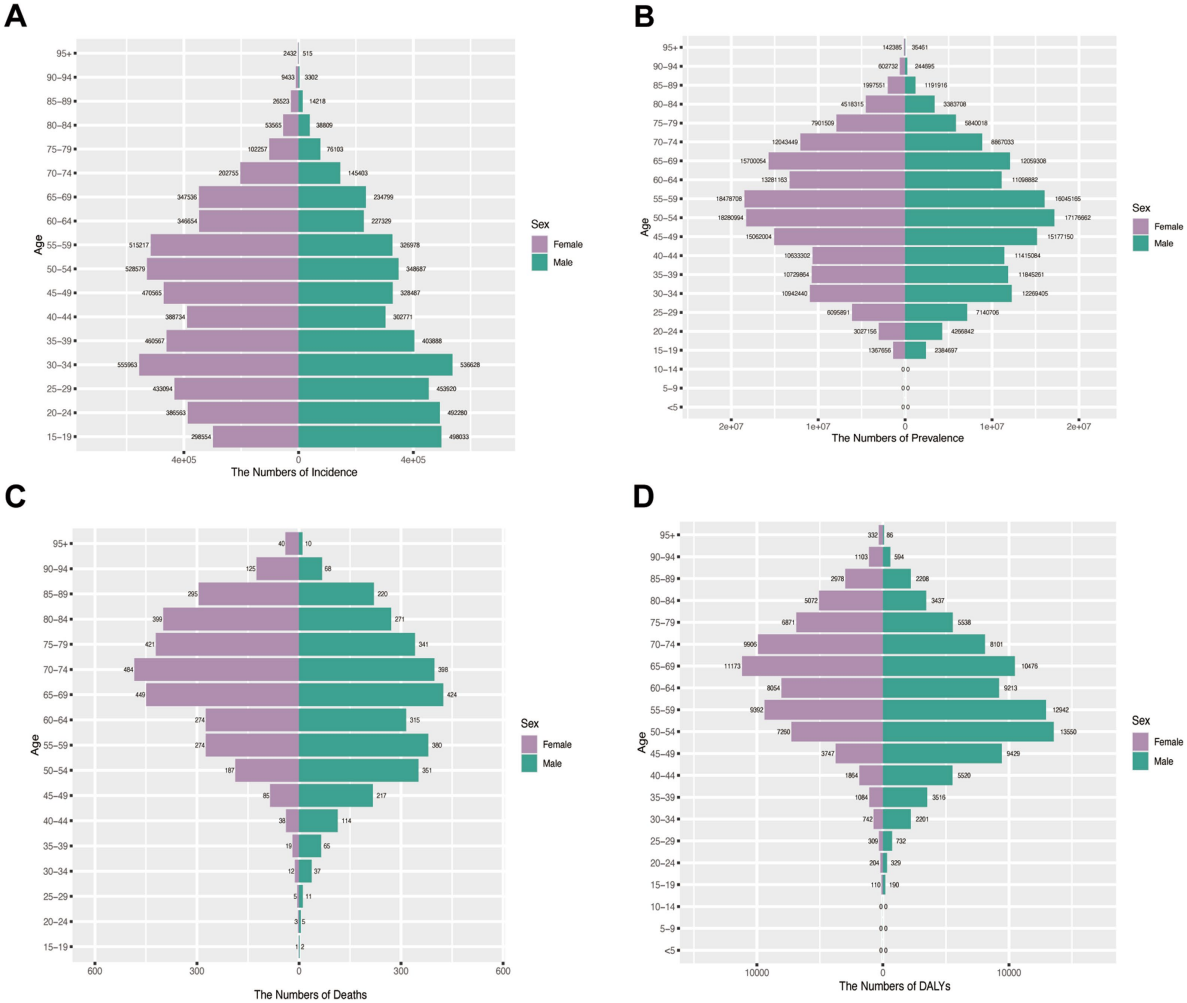
Assesses sex- and age-specific variations in burden across four metrics in China (2021). **(A)** Incidence; **(B)** Prevalence; **(C)** Mortality; **(D)** DALYs.

Incidence patterns: Peak shifted from 20 to 24 (1990) to 30–34 (2021) for both sexes. Females maintained higher incidence rates across most age groups except 15–24 years in 1990, whereas by 2021 males exhibited greater incidence below age 29 with female predominance persisting in older cohorts (Figures 6A, 7A).

Prevalence trends: The 35–39 age group represented the prevalence peak for both sexes in 1990. Male predominance <55 years (1990) shifted to female predominance >50 years (2021) (Figures 6B, 7B).

Mortality and DALY burden: Female predominance in older ages (≥60 in 1990; ≥65 in 2021), peak at 70–74 years. Male peak earlier. DALY patterns mirrored mortality: female peak shifted from 55 to 59 (1990) to 65–69 (2021); male peak at 50–54 (Figures 6C,D, 7C,D).

**Figure 8:**
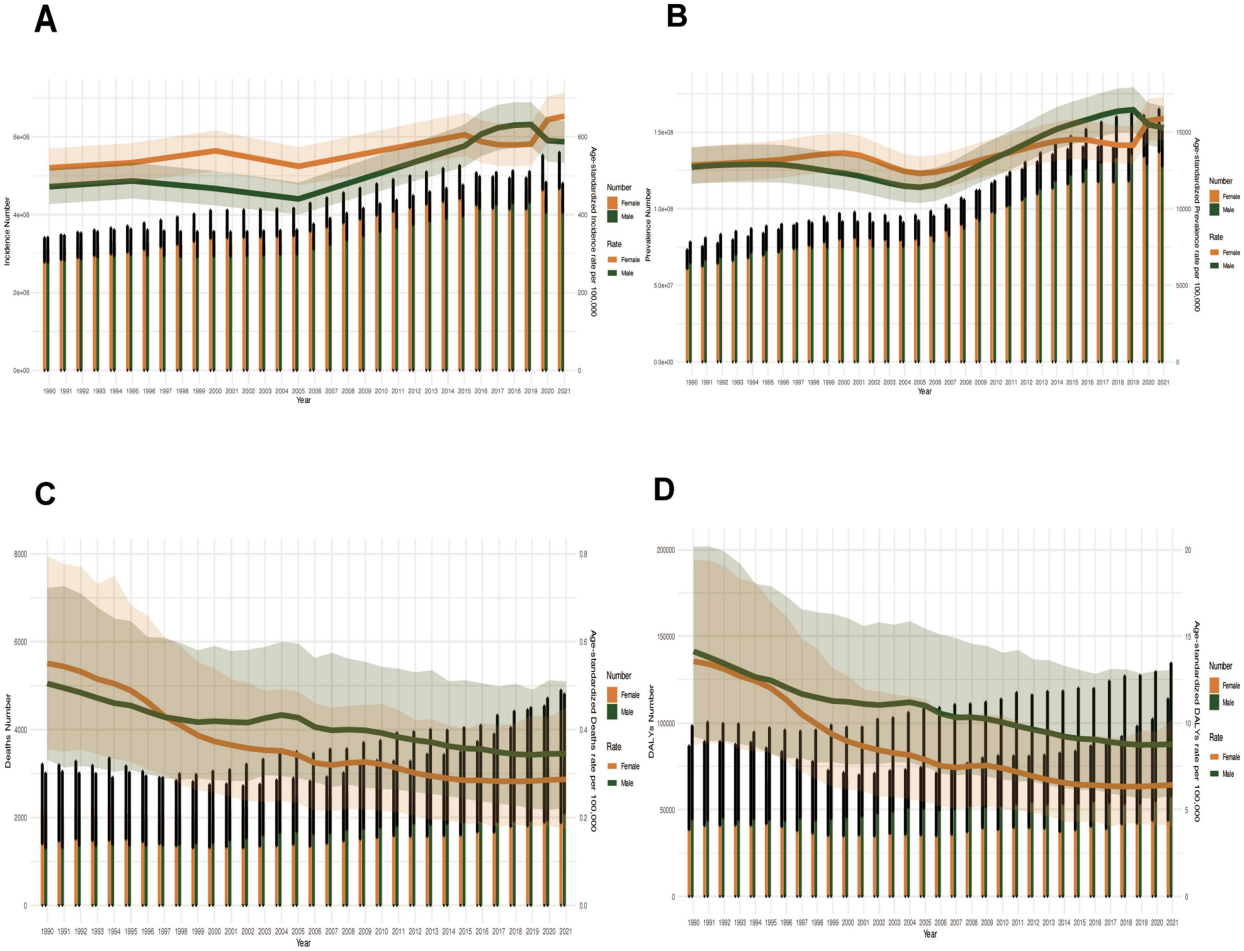
Sex-specific disparities in disease burden metrics across all age groups in China (1990–2021). **(A)** Incident cases and ASIR; **(B)** Prevalent cases and ASPR; **(C)** Death cases and ASMR; **(D)** DALYs counts and ASDR. Bar charts represent counts; lines represent age-standardized rates.

Age-standardized rates: Female ASIR dominated except 2016–2020 (peak female ASIR in 2021). ASPR fluctuated intersectingly. Post-1996, females had significantly lower ASMR and ASDR than males (Figures 8A–D).

## Discussion

This study systematically analyzed the burden of metabolic dysfunction-associated steatotic liver disease (MASLD)-related chronic liver diseases (CLD) and cirrhosis in China and globally from 1990 to 2021 using Global Burden of Disease (GBD 2021) data, focusing on incidence, prevalence, mortality, and disability-adjusted life years (DALYs). The results revealed distinct temporal trends and demographic patterns.

In China, age-standardized incidence (ASIR) and prevalence rates (ASPR) showed a temporary decline during 2000–2005 (*p* < 0.05), followed by a rapid increase after 2005. In contrast, global rates exhibited continuous growth throughout the study period. While China achieved significant reductions in MASLD-related mortality and disease burden, global progress remained limited.

Age-specific analysis demonstrated persistently high incidence among young adults and elevated disease burden among elderly populations, who accounted for most deaths and DALYs. Sex disparities were evident, with women facing higher risks after age 50 and young men showing rapidly increasing incidence rates.

These findings highlight China’s “dual burden” of MASLD: rising incidence in youth suggests early-onset metabolic risks, while high mortality in the elderly underscores the need for better complication management. Although China outperformed global trends in reducing mortality and DALYs, persistently increasing incidence and prevalence rates indicate ongoing challenges.

The sharply rising incidence/prevalence rates observed in China, particularly the accelerating disease occurrence among younger demographics, provide robust evidence for rapidly advancing urbanization and lifestyle shifts acting as principal drivers, with this impact potentially amplified within the Chinese context (24, 25).

Notably, China has achieved significant reductions in MASLD-related mortality and disease burden, contrasting with limited global progress. This improvement likely reflects substantial public health advancements over the past three decades. While global data provide valuable insights into overall disease burden, they may not adequately represent specific national or regional contexts.

Comparative analyses reveal lower MASLD prevalence in high-income regions including Asia-Pacific, North America, Western Europe, and Australasia (26).

Our findings reveal significant sex-based differences in disability-adjusted life years (DALYs) attributable to MASLD. Overall, men exhibit higher DALYs than women, likely due to the absence of estrogen’s protective effects and a higher prevalence of metabolic risk factors, including increased visceral fat accumulation, insulin resistance, poorer dietary habits, and suboptimal health management. However, in our study, women aged >65 years carried a more substantial disease burden than their male counterparts. This aligns with prior research suggesting that hormonal transitions, particularly during menopause, may accelerate MASLD progression in older women (27). However, this biological mechanism likely interacts with additional systemic barriers that disproportionately affect this population, particularly within rural healthcare settings such as Western China. Critically, geographic disparities across China’s regions substantially modulate disease burden, compounded by gender-specific healthcare disparities. Women in underserved areas confront compounded vulnerabilities: (1) Cultural and Socioeconomic Barriers: Traditional prioritization of male healthcare needs, coupled with financial constraints, frequently delays female presentation for symptomatic liver disease and restricts adherence to regular monitoring and pharmacotherapy; (2) Healthcare Engagement Barriers: Limited health literacy and reduced screening uptake contribute to diagnostic delays; (3) Comorbidity Management Deficits: Concomitant conditions often remain under-recognized and undertreated. Infrastructure Inequities further exacerbate these disparities: Hepatology services and tertiary care centers remain predominantly concentrated in eastern metropolises (e.g., Beijing, Shanghai). Consequently, elderly women in rural and western regions experience profound deficits in accessing guideline-concordant MASLD management and specialized cirrhosis care throughout the disease continuum.

These findings underscore the critical role of sex in MASLD epidemiology and highlight the need for sex-specific health management and prevention strategies to mitigate the global burden of MASLD and its complications.

In China, elderly individuals (≥70 years) constitute the primary demographic group bearing the highest burden of MASLD-related mortality and disability-adjusted life years (DALYs), underscoring the critical imperative for effectively managing MASLD complications, such as cirrhosis and hepatocellular carcinoma (HCC), to interrupt this well-established disease trajectory. Given the rapidly aging population structure, these findings necessitate urgent action: (1) optimizing metabolic risk management within this group; (2) enhancing the screening and management of cirrhosis and its complications (including variceal hemorrhage and hepatic encephalopathy); and (3) developing integrated liver disease management models incorporating cardiovascular disease (CVD) prevention and control strategies, as CVD represents the leading cause of death among MASLD patients (28).

Notably, we observed a shift in the peak age of MASLD incidence from 20 to 24 years in 1990 to 30–34 years in 2021. This temporal transition of the age peak may reflect: (1) the delayed manifestation of cumulative childhood obesity effects; (2) prolonged exposure to adverse lifestyle factors during early adulthood; or (3) improvements in diagnostic awareness or evolving diagnostic methodologies—a possibility warranting further investigation. Nevertheless, the established recognition of childhood obesity as a key risk factor (29), underscores the critical necessity of obesity interventions beginning in early life to mitigate the rising prevalence of MASLD among younger age cohorts.

### Policy and clinical implications

From 1990 to 2021, NAFLD prevalence has surged dramatically, yet it remains underprioritized in global and national public health strategies (30). This lack of focus has led to low disease awareness, even among high-risk populations and healthcare providers. Given MASLD’s strong links to metabolic syndrome, cardiovascular disease, and liver complications, integrating MASLD screening into routine metabolic health assessments could improve early detection and intervention.

Current management relies heavily on lifestyle interventions, given NAFLD’s bidirectional relationship with metabolic syndrome. Key findings include: Weight loss of 5–7% significantly reduces liver fat. Weight loss exceeding 10% may even reverse fibrosis (31).

While no FDA/EMA-approved MASLD -specific therapies exist, several repurposed drugs show promise: Pioglitazone (improves insulin sensitivity); Vitamin E (antioxidant effects in non-diabetic MASH); GLP-1 agonists (weight loss and metabolic benefits); SGLT-2 inhibitors (cardio-metabolic and potential hepatic benefits) (32, 33). These agents, currently used for diabetes and obesity, may soon play a role in personalized MASLD treatment regimens. However, further large-scale randomized trials are needed to confirm their efficacy in halting disease progression.

Implementing targeted interventions for youth is crucial for primary prevention. Specific policy actions should include: (1) integrating metabolic risk factor assessment (e.g., BMI, waist circumference, blood pressure) into routine school health screenings to identify high-risk adolescents early; (2) developing and promoting accessible digital health tools (apps/platforms) tailored for adolescents to encourage healthy eating and physical activity; (3) incorporating comprehensive nutrition education and healthy lifestyle modules into school curricula; (4) enacting regulations to restrict marketing of unhealthy foods and beverages targeted at children and adolescents; and (5) improving community infrastructure to increase access to safe spaces for physical activity.

For managing the complex comorbidities prevalent in the elderly MASLD population, establishing Integrated Cardiometabolic-Liver Clinics represents a promising framework. These specialized clinics would bring together hepatologists, endocrinologists/diabetologists, cardiologists, nutritionists, and other relevant specialists within a single, coordinated service. The model would facilitate comprehensive assessment of cardiovascular, metabolic, and liver health simultaneously, enabling truly individualized management plans that address all facets of the patient’s condition. This integrated approach aims to reduce fragmented care, improve patient adherence, optimize resource utilization, and ultimately lead to better health outcomes for this vulnerable group.

Given the growing arsenal of lifestyle interventions and pharmacotherapies, high-burden countries must adopt evidence-based approaches to curb MASLD’s escalating impact. A comprehensive management strategy—combining prevention, early detection, and accessible treatment—could be pivotal in reducing both MASLD prevalence and its complications.

## Limitations of the study

This study presents the first systematic comparison of MASLD disease burden between China and the global population from 1990 to 2021, providing valuable evidence for health policy-making worldwide. However, several important limitations should be noted. First, the accuracy of GBD estimates is inherently constrained by the quality and completeness of underlying data sources, particularly in resource-limited settings and economically disadvantaged populations where underdiagnosis is prevalent due to inadequate screening systems.

This limitation is particularly acute in rural China, where fundamental barriers to MASLD diagnosis compound underdetection: (1) Limited Specialist Access: Scarce availability of specialized healthcare facilities and gastroenterologists with expertise in identifying and confirming steatotic liver disease diagnoses; (2) Economic Constraints: Financial burdens disincentivize patients from pursuing evaluation for frequently asymptomatic liver conditions; and (3) Infrastructural Deficits: Underutilization of essential imaging modalities (e.g., ultrasound) for systematic screening within primary care settings. Collectively, these factors drive significant underdiagnosis, which systemically compromises epidemiological accuracy by artificially suppressing reported incidence rates.

Particularly concerning is that underdiagnosis, especially in resource-constrained settings, constitutes a source of systematic bias. This may lead to a systematic underestimation of the burden of MASLD. Such bias thereby risks undermining the reliability of international comparisons of disease burden. Consequently, future research should prioritize the development of standardized screening tools, with the aim of mitigating the impact of underdiagnosis. Second, while the global-level aggregation offers a comprehensive overview, it may mask significant regional variations in disease burden attributable to differences in socioeconomic development, genetic predisposition, healthcare infrastructure, and environmental exposures. Third, despite employing active case-finding methodologies, the true burden of MASLD is likely underestimated given the typically asymptomatic nature of early-stage disease and the lack of routine screening protocols in most healthcare systems. Fourth, the analysis did not account for competing risks, such as deaths from cardiovascular disease (CVD). This omission is particularly relevant because CVD mortality can reduce the observed occurrence of MASLD-related deaths—e.g., patients may die from CVD before developing fatal MASLD complications—potentially leading to an underestimation of MASLD-specific mortality risk. Future research should incorporate competing risks models to provide more accurate burden estimates. These limitations suggest that while our findings provide important global benchmarks, they should be interpreted with caution and supplemented with local epidemiological data for precise policy formulation. Future research should prioritize strengthening surveillance systems in understudied regions and developing standardized screening approaches to improve case detection (17, 19).

## Conclusion

This study demonstrates a progressive increase in the prevalence of MASLD over the past three decades. Chinese national surveillance data confirm a critical imperative for precision metabolic risk mitigation targeting adolescents and young adults. The observed epidemiological transition—characterized by a peak in incidence among younger populations and a concentration of deaths in older adults—calls for integrated strategies including early-life lifestyle interventions and comprehensive management frameworks for comorbidities in the elderly.

These findings provide critical evidence to guide the establishment of disease-specific targets, assess epidemiological trends, and allocate resources for the prevention, diagnosis, and management of MASLD in China and worldwide. Effective public health strategies—including lifestyle interventions and improved healthcare accessibility—are essential to mitigate the future burden of this disease.

## Data Availability

Publicly available datasets were analyzed in this study. This data can be found at: https://vizhub.healthdata.org/gbd-results/.

## Footnotes

^1^https://vizhub.healthdata.org/gbd-results
